# Penetrating Keratoplasty in Congenital Glaucoma

**DOI:** 10.3390/jcm12196276

**Published:** 2023-09-29

**Authors:** Bilge Batu Oto, Nevbahar Tamçelik, Ercüment Bozkurt, Ceyhun Arici, Oğuzhan Kılıçarslan, Busenur Gönen, Hacı Uğur Çelik

**Affiliations:** 1 Department of Ophthalmology, Cerrahpaşa Medical Faculty, Istanbul University-Cerrahpaşa, 34098 Istanbul, Turkey; 2 Tamcelik Glaucoma Center, 34394 Istanbul, Turkey; 3 Department of Ophthalmology, Batı Göz Merkezi, 34662 Istanbul, Turkey; 4 Department of Ophthalmology, Ayancık State Hospital, 57400 Sinop, Turkey; 5 Department of Ophthalmology, Zonguldak Atatürk State Hospital, 67030 Zonguldak, Turkey; 6 Flaum Eye Institute, School of Medicine and Dentistry, University of Rochester, New York, NY 14627, USA

**Keywords:** congenital glaucoma, penetrating keratoplasty, aniridia, Axenfeld–Rieger anomaly, primary congenial glaucoma

## Abstract

Background: Childhood glaucoma is one of the most common causes of corneal opacity in childhood and is associated with various pathological corneal changes, including corneal enlargement, corneal clouding, and edema. Congenital glaucoma (CG) may cause a decrease in vision outcomes due to corneal opacity or clouding, which is often associated with stimulus deprivation amblyopia. Therefore, to create a balance between preventing amblyopia and sustaining corneal clearance, patients with CG can be managed with early penetrating corneal transplantation surgery along with advanced glaucoma management. Aim: To investigate the graft survival rate and factors affecting graft survival in patients with congenital glaucoma who underwent penetrating keratoplasty (PKP). Study Design: Cross-sectional. Materials and Methods: Patients with congenital glaucoma who underwent PKP were retrospectively evaluated. The associations between age, corneal diameter, presence of ocular comorbidities, concurrent ocular surgeries with corneal graft, and visual outcomes were assessed. Results: Among the 30 eyes enrolled in the study, 6 (20%) had aniridia, 6 (20%) had Axenfeld–Rieger syndrome, and 18 (60%) were diagnosed with primary congenital glaucoma. Graft survival rates were 66.6% and 63.33% at 12 and 24 months, respectively. At the end of the follow-up, the overall graft survival rate was 60%. Statistical significance was observed between patient age at the time of surgery and graft failure (*p* = 0.02). Graft failure was associated with a younger patient age. Functional vision was achieved in 53.3% of patients. Conclusions: The management of congenital glaucoma and its corneal complications is a delicate issue that requires great effort. PKP in congenital glaucoma was moderately successful in the present study. To provide functional vision, PKP could be the treatment of choice.

## 1. Introduction

Childhood glaucoma, a developmental anomaly of the anterior segment, is one of the most common causes of corneal opacity in childhood [1]. It is associated with various pathological corneal changes, including corneal enlargement (buphthalmos) related to increased intraocular pressure (IOP), increased corneal thickness, corneal clouding, and edema caused by Descemet’s membrane tears (Haab’s striae) [2,3,4]. Congenital glaucoma (CG) may cause a decrease in vision outcomes due to glaucomatous optic neuropathy and changes in the cornea in cases of IOP control failure. Corneal opacity or clouding is often associated with stimulus deprivation amblyopia [5].

To create a balance between preventing amblyopia and sustaining corneal clearance, patients with CG can be managed with early penetrating corneal transplantation surgery along with advanced glaucoma management [1,6]. The success rate of penetrating keratoplasty (PKP) depends on multiple preoperative or postoperative risk factors for childhood glaucoma. Patient age, host and graft corneal diameter, concurrent glaucoma, and lens surgeries have been noted as controversial prognostic factors for early or long-term graft survival in several studies [7].

In this study, we evaluated a large case series of patients diagnosed with different types of CG who required PKP. We analyzed primary etiologies, anatomic corneal features, additional surgeries, and other factors with respect to their effects on graft survival.

## 2. Materials and Methods

In this retrospective study, 28 CG patients diagnosed with primary congenital glaucoma (PCG), aniridia, and Axenfeld–Rieger syndrome who underwent corneal transplantation were enrolled. Thirty eyes that underwent PKP were included in the transplant survival analysis and were the focus of this study. The records from 2000 to 2020 were screened at the Tamçelik Glaucoma Center, Istanbul, Turkey. The study followed the tenets of the Declaration of Helsinki and was approved by the institutional ethics committee (07/10/2020-68125). Subjects, or their legal guardians in applicable cases, signed a consent form before participation.

Congenital glaucoma diagnoses were recorded from the charts. The diagnosis was made based on the following ocular findings: elevated IOP (>18 mmHg), buphthalmos, Haab’s striae, corneal stromal edema, and glaucomatous optic disc change, which had been present at birth or after birth. Patients with childhood glaucoma or juvenile onset were excluded. Medical records were screened for age, sex, surgical indications, primary diagnosis, procedure side, associated surgical procedures, preoperative or postoperative glaucoma, preoperative and last-visit vision, graft clarity, time to graft failure, major postoperative complications and rejection episodes, and outcomes. The associations between age, ocular pressure at the time of surgery, corneal diameter, the presence of ocular comorbidities, and concurrent ocular surgeries with corneal graft outcomes were evaluated. Visual outcomes were also assessed.

Proper control of IOP was provided for all patients, and only patients with controlled IOP (IOP ≤ 18 mmHg) underwent PKP. In patients with uncontrolled IOP, IOP control was achieved with topical medical treatment IOP or surgical treatment before proceeding with PKP. Surgical treatment options to control IOP prior to PKP included trabeculectomy with or without Mitomycin C or Ahmed glaucoma valve implantation.

Corneal graft failure was defined as the presence of corneal central opacity that was no longer compatible with good visual function. Visual function was recorded as the best visual acuity measured during the first and last visits. In children with insufficient verbal or cognitive skills, the ability to fixate on and follow visual targets was measured. Low vision evaluation was classified into light perception, hand movement, and finger count. Light perception was recorded as 0.002 on the decimal scale and 2.70 on the logarithm of the minimum angle of resolution (LogMAR) scale. Hand movement was recorded as 0.0063 on the decimal scale and 2.20 on the LogMAR scale [8].

### 2.1. PKP Surgical Technique

All surgeries were performed under general anesthesia by an experienced corneal transplant surgeon. Corneas were preserved in Optisol (Bausch & Lomb Inc., Rochester, NY, USA, EUA). Trephination was manual, with a difference in diameter between the recipient and donor between 0.50 and 0.75 mm. The transplant was sutured using 16–24 interrupted mononylon 10-0 sutures. Subconjunctival antibiotics and steroids were applied at the end of the procedure, and the occlusion was maintained for 24 h.

Topical antibiotics and corticosteroids were administered postoperatively. Sutures were removed between 6 and 8 weeks postoperatively in patients under 2 years of age, between 8 and 12 weeks in patients aged 3–5 years, and up to 1 year postoperatively in patients over 5 years. Optical correction with spectacles and amblyopia treatment was initiated as soon as possible. All loose sutures were removed when observed, and complications were promptly treated.

Graft rejection was determined when corneal clarity was irreversibly lost in the absence of other potential causes. When multiple PKPs were performed on a patient, the first surgery was considered for analysis. Regular visits were scheduled in the glaucoma clinic after the surgery. When an optimal exam was not feasible, examinations were performed under general anesthesia. IOP measurements below 18 mmHg, with or without medication, were considered successful IOP management. A clear graft was defined as the absence of corneal edema, which provided a clear view of the underlying anterior segment chamber details. The graft survival time was defined as the period from PKP to the day when the diagnosis of graft failure occurred or was last recorded during postoperative clinical visits. All patients were followed up for at least 12 months after the surgery.

### 2.2. Statistical Analysis

Statistical analyses were performed using IBM SPSS Statistics software (version 20.0, Chicago, IL, USA). The Kaplan–Meier and Cox models for survival were used to evaluate the correlation between graft survival time and corneal opacity, diagnosis, age at surgery, and rejection. Survival curves were compared using the Mantel–Cox and Breslow tests. Specifically, for patients with congenital opacity, the effects of age during surgery on graft survival were evaluated. A Cox model was used to simultaneously assess the correlation between graft survival time and corneal opacity, diagnosis, rejection, and age at surgery. Descriptive analyses were presented using means and standard deviations (SDs), as the variables were normally distributed. T-tests were used to compare two independent groups with normal distribution. Pearson’s correlation was used to assess the relationship between the time until rejection and the age at which the PKP was performed. Situations where the *p*-value was below 0.05 were evaluated as statistically significant results.

## 3. Results

In the study, 30 eyes of 28 patients were included: 13 patients (46.42%) were male and 15 (53.57%) were female. Of the sample, six eyes (20%) had aniridia, six (20%) had Axenfeld–Rieger syndrome, and eighteen (60%) were diagnosed with PCG. At the time of PKP, the median age was 84 [138] months (range, 24–600 months). The mean follow-up time was 116.43 ± 72.19 months (minimum 11, maximum 246). The average time to graft failure after initial PKP was 22.25 ± 39.61 months (range 3–120 months). Two patients underwent concomitant lensectomy with PKP, and one developed graft failure 6 months after PKP. This patient was regrafted, and the cornea remained clear during the three-year follow-up period.

**Figure 1:**
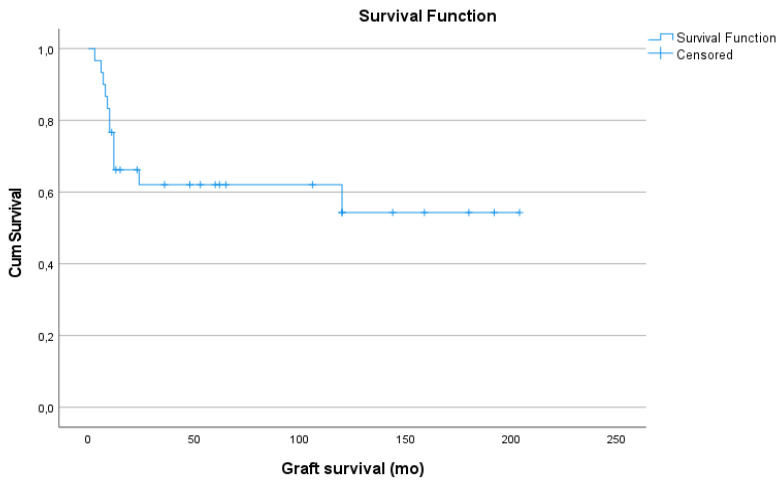
Kaplan–Meier survival analysis of graft survival.

Graft failure occurred after PKP in 12 of 30 eyes (40%); among these 12 eyes, 5 underwent regrafting, and 3 regrafted corneas had graft failure again. Failures occurred mainly within the first year after PKP (10 of 12 grafts failed within the first year of PKP). Graft survival rates were 66.6% and 63.33% at 12 and 24 months, respectively (Figure 1). At the end of the follow-up, the overall graft survival rate was 60%. Table 1 shows the primary diagnoses of failed grafts.

**Figure 2:**
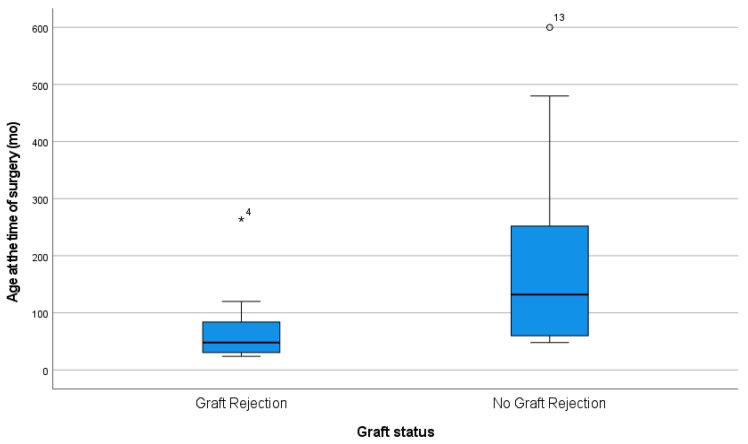
Relation of age at the time of surgery and graft rejection.

Statistical significance was observed between patient age at the time of surgery and graft failure (*p* = 0.02). Graft failure was associated with a younger patient age (Figure 2). However, no statistical relationship was established between patient age at the time of surgery and the time to rejection (*p* = 0.49).

The median corneal diameter at the time of PKP was 13 mm (range, 12–16 mm). No statistical relationship was observed between corneal diameter and graft failure (*p* = 0.46).

None of the patients underwent further glaucoma surgeries after corneal grafting during the follow-up period. The final mean IOP was 16.79 ± 3.60 mmHg (range 10–25 mmHg). The average glaucoma medication used to control IOP was 1.83 ± 1.14 per eye (range 1–3). The antiglaucomatous medications used for each patient according to the characteristics of the patient and the type of glaucoma included β blockers, α agonists, carbonic anhydrase inhibitors, and prostaglandin analogues.

Before PKP, none of the patients had functional vision. After PKP, a visual acuity of 20/400 or better was achieved in 16/40 (53.3%) eyes. In the remaining 14 eyes, vision of light perception or counting fingers could be achieved (Table 2).

Fourteen eyes (46.66%) underwent Ahmed glaucoma valve (AGV) surgery before PKP. Among the fourteen eyes with AGV, graft failure occurred in six eyes (42.85%). There was no statistical relationship between the presence of AGV and graft failure (Pearson’s chi-squared test, *p* = 0.76).

## 4. Discussion

The success of PKP in treating childhood glaucoma depends on many preoperative and postoperative risk factors. Patient age at the time of corneal surgery has been one of the most discussed factors in graft survival [9,10]. Many reports suggest that young age during PKP is related to poor prognosis. The high frequency of different spectra of anterior segment dysgenesis in younger patients was proposed as one of the reasons for worse graft survival. Corneal diameter has been suggested as a prognostic factor for graft failure. A smaller corneal diameter is possibly associated with poorer graft survival due to reduced exposure to transplanted endothelial cells [11]. Other ocular surgeries, along with PKP, have been widely investigated for CG. In several studies, concurrent glaucoma or lens surgery was found to be a negative prognostic factor for early or long-term graft survival in several studies [7,9].

Corneal pathologies associated with CG are well-known but rare indications for PKP and have commonly shown a poor prognosis. The graft survival rates were 66.6% and 63.33% at 12 and 24 months, respectively, and the overall graft survival rate was 60% in our study group. Ariyasu et al. reported a graft survival rate of 66.6% (six of nine grafts) with a mean 30-month follow-up [12]. Additionally, Toker et al. reported that in buphthalmic eyes that also included secondary pediatric glaucoma, the success rate of PKP was 75% for an average follow-up period of 57.1 ± 49.1 months; however, the rate of having a clear graft after the first PKP was calculated as 40% [13]. The lower success rate in our study compared to the previous study may be due to the longer follow-up period, and our success rate was evaluated based on the results of the initial PKP. The reasons for poor prognosis may include the age at the time of keratoplasty and the presence of previous glaucoma surgeries.

Graft failure was found to be associated with younger age at the time of PKP in our study, which included patients with CG. Various studies have shown inconsistent graft survival rates related to age at transplantation. Some studies have reported that corneal transplantation in younger patients is associated with a higher incidence of graft failure [9,14], whereas others found no significant difference in graft survival based on patient age [7,11,15]. Better analysis could be performed through larger-scale studies in which patients were sub-grouped according to age.

Several studies have reported that the presence of glaucoma alone [16,17,18,19] influences graft survival, even if IOP is under control [15]. Di Zazzo et al. stated that CG has a worse prognosis than other reasons for graft failure following pediatric PKP [20]. Therefore, handling patients with CG who require PKP is a delicate issue.

Previous glaucoma surgeries have been associated with a higher incidence of graft failure [16,21]. Considering that all CG patients in our study underwent previous glaucoma surgery, an overall graft survival of 60% at the end of the follow-up period was considered a successful result.

It is known that concurrent glaucoma surgery with PKP is associated with poor graft survival [7,13]. In this recent study, all patients had good IOP control before PKP. In eyes with poor IOP control, PKP was deferred and glaucoma surgery was prioritized. Fourteen eyes (46.66%) underwent AGV implantation before PKP. Corneal opacity was present in these patients before AGV implantation and did not occur as a complication of AGV surgery. Proper control of IOP prior to PKP may be the major reason for graft survival.

A smaller recipient corneal diameter has been found to be more related to graft failure in various studies [11,13]. Although there are no existing data in the literature, we observed that higher trephine/punch rates resulted in better graft survival. We believe that traction on the cornea is decreased in large grafts. Therefore, trephination was made with a difference in diameter between 0.50 and 0.75 mm in the recipient and donor. No statistical relationship between corneal diameter and graft failure was observed in our study because all patients had corneal diameters >12 mm. The study group comprised patients with CG and buphthalmos.

Visual prognosis after PKP in congenital glaucoma patients was reported to be poor [12,22]. A previous study reported no significant difference in visual acuity between pre- and post-PKP in patients with CG and concluded that 47% of patients maintained the same visual acuity [23]. Our study revealed that even patients with light perception could gain functional vision (≥20/400). Visual improvement was achieved in 53.3% of patients after PKP. Regardless of age, PKP could be the treatment of choice in patients with light perception.

Graft failure occurred mostly within the first year of PKP; the time to graft failure was independent of patient age.

The management of CG and its corneal complications is a delicate issue that requires great effort. With PKP, functional vision could be achieved even in patients with only light perception.

In conclusion, there are various factors influencing the graft survival following PKP. In our study, we observed that younger age was the major factor affecting graft survival in patients with CG. Larger-scale studies may identify the presence of other factors that affect graft survival.

## Figures and Tables

**Table 1:** Graft survival according to primary diagnosis.

Diagnosis	Graft Failure (*n*)	Graft Survival (*n*)	Total (*n*)
**Axenfeld–Rieger Anomaly**	3	3	6
**Aniridia**	3	3	6
**PCG**	6	12	18

*n* number, PCG primary congenital glaucoma.

**Table 2:** Visual acuity before and after PKP.

Visual Acuity	Before PKP (Number of Patients)	After PKP (Number of Patients)
**LP-CF**	26 *	14
**20/400**	-	11
**20/200**	-	2
**20/100**	-	2
**20/63**	-	1

PKP: penetrating keratoplasty, LP-CF: light perception/counting fingers. * VA assessment was not possible preoperatively in 4 patients due to young age.

## Data Availability

Research data could be shared upon request to the corresponding author.
